# Formate Production from Simulated Quasi‐Flue Gas Combining a Molecular Catalyst and a Modified Electrode

**DOI:** 10.1002/cssc.202500392

**Published:** 2025-06-13

**Authors:** Yutzil Segura‐Ramirez, Maria Gomez‐Mingot, Marc Fontecave, Carlos M. Sánchez‐Sánchez

**Affiliations:** ^1^ Laboratoire de Chimie des Processus Biologiques Collège de France UMR 8229 CNRS Sorbonne Université PSL Research University 11 Place Marcelin Berthelot 75005 Paris France; ^2^ Sorbonne Université CNRS, Laboratoire Interfaces et Systèmes Electrochimiques (LISE) 4 Place Jussieu 75005 Paris France

**Keywords:** CO_2_ electroreduction, immobilized imidazolium, impurity tolerance, molecular electrochemistry, simulated flue gas

## Abstract

Molecular metal complexes form an important class of catalysts for the electroreduction of CO_2_ (CO_2_RR) to carbon monoxide (CO) or formic acid (HCOOH), key processes in the context of the requested exploration of novel sources of carbon, alternative to fossil fuels. Research studies are most generally carried out with pure gas streams of CO_2_, while the available real sources of CO_2_ are gases coming out from industrial plants and containing a low share of CO_2_, and a great diversity of impurities including nitrogen and sulfur oxides. Herein, it is shown that a molecular catalyst, [Rh(bpy)(Cp*)Cl]Cl (bpy = bipyridine, Cp* = pentamethylcyclopentadienyl), catalyzes CO_2_RR to formic acid using a quasi‐flue gas (5–10% CO_2_ and 100 ppm NO_2_ or 50 ppm SO_2_) with substantial selectivity. This is made possible thanks to the modification of the cathode surface with a positively charged imidazolium layer, which greatly favors CO_2_RR over competing reactions, proton, NO_2_, and SO_2_ reductions. These results highlight the potential of combining molecular catalysis and electrode surface modification for the electroreduction of diluted CO_2_ without prior carbon capture or purification.

## Graphical abstract

A modified electrode with a positively charged imidazolium layer, together with a molecular Rh complex in solution, achieves high selectivity for CO_2_ conversion to HCOOH from a simulated industrial flue gas stream containing diluted CO_2_ and impurities such as NO_2_ and SO_2_.

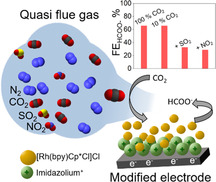

## 1 Introduction

Carbon dioxide (CO_2_) emissions contributing to the global greenhouse effect come from a variety of sources, cement and steel production, coal combustion plants, as well as waste incinerators, being some of the most relevant individual contributors.^[^ ^1^ ^]^ Thus, addressing the conversion and recycling of the annual millions of tons of CO_2_ expelled by those specific sources represents an effective strategy to limit CO_2_ adverse effects. CO_2_ electroreduction^[^ ^2^ ^]^ (CO_2_RR) is an environmentally friendly approach if fueled with low‐carbon electricity. It implies complex multielectron and multiproton reactions usually accompanied by competitive hydrogen evolution reaction (HER) and thus necessitates selective and efficient catalysts.^[^ ^3^, ^4^, ^5^, ^6^, ^7^, ^8^, ^9^ ^]^ As shown from reports based on data collected by the U. S. Environmental Protection Agency in 2022,^[^ ^10^ ^]^ the majority of industrial plants (refineries, power, metallurgy, and waste incineration plants) release flue gases with low CO_2_ concentration (4–25% v/v). Furthermore, depending on the industrial process at work, the flue gas can contain water, oxygen, and pollutants such as particulate matter, amines, aromatic hydrocarbons, halocarbons, heavy metals, as well as nitrogen oxides (NO_*x*_), sulfur dioxide (SO_2_), hydrogen sulfide (H_2_S), or hydrogen cyanide (HCN).^[^ ^11^, ^12^ ^]^ To date, most research on CO_2_RR is conducted in ideal laboratory conditions, using 100% pure CO_2_ gas as a reactant into electrolyzers, to avoid any deleterious effect due to both low concentration of CO_2_ and the presence of traces of impurities.^[^ ^12^, ^13^, ^14^, ^15^ ^]^ Initially, carbon capture and utilization technologies based on CO_2_ purification through CO_2_‐capturing reactions using amines, which have been already industrially implemented,^[^ ^16^, ^17^, ^18^, ^19^ ^]^ were considered as a suitable strategy to overcome the limitations of working with diluted CO_2_ streams. However, this strategy is still energy‐intensive and expensive due to the multiple steps involved.^[^ ^20^, ^21^ ^]^ Thus, the direct utilization of diluted CO_2_ from flue gas without capture and purification represents a potential alternative.^[^ ^22^, ^23^, ^24^, ^25^, ^26^, ^27^ ^]^

Molecular catalysts for CO_2_RR have concentrated a great deal of attention for direct CO_2_ conversion since they reach a very high product selectivity and they can be finely tuned via synthetic modifications of the ligands,^[^ ^28^ ^]^ as well as variations of the metal ions present within the molecular catalyst.^[^ ^29^, ^30^, ^31^, ^32^, ^33^, ^34^ ^]^ This can be achieved for example using molecular catalysts with CO_2_ capturing ability, as illustrated by the work of Ishitani and collaborators.^[^ ^25^, ^26^, ^27^ ^]^ They have modified the coordination sphere of a Re(I) complex by adding a deprotonated triethanolamine as a ligand and this catalytic complex showed high selectivity for CO production at low concentrations of CO_2_ such as 10% and even 1%, with Faradaic efficiencies for CO of 95% and 85%, respectively.^[^ ^25^, ^26^, ^27^ ^]^ We have recently proposed an alternative strategy to improve molecular catalysts’ performance for CO_2_RR by tuning the local electric field at the electrode/electrolyte interface either using ionic liquids as electrolyte^[^ ^35^, ^36^ ^]^ or immobilizing charged organic molecules such as imidazolium on the electrode surface.^[^ ^37^ ^]^ This latter approach based on imidazolium‐modified electrodes was successfully scaled up operating at industrially relevant conditions (100 mA cm^−2^) on a gas diffusion electrode in a flow cell type reactor. The effect of the imidazolium layer was specifically assigned to its capacity to interact with the soluble molecular catalyst, namely, the Rh complex ([Rh(bpy)(Cp*)Cl]Cl; bpy = bipyridine, Cp* = pentamethylcyclopentadienyl),^[^ ^38^ ^]^ via π^+^–π interactions between the imidazolium cation and the reduced bipyridine ligand, which resulted in CO_2_RR overpotential decrease and partial HER suppression, as supported by density funtional theory (DFT) calculations.^[^ ^37^ ^]^ This complex is an excellent model molecular catalyst for studying the competition between CO_2_RR to formic acid and HER, as it is active for both reactions.^[^ ^38^, ^39^, ^40^ ^]^ It was thus tempting to push further the electrode surface modification approach to evaluate its potential for maintaining CO_2_RR selectivity even converting diluted CO_2_ streams, as this approach was very little explored so far. For example, a recent report exploited an organic hydrophobic polymer (poly(4‐vinylpyridine)) for modifying carbon black nanoparticles deposited into a gas diffusion layer, used as the electrode material, and showed enhanced CO generation (Faradaic efficiency 90%) by electrolysis of dilute CO_2_ (10% v/v) using a Co complex as a catalyst.^[^ ^41^ ^]^ Here, we actually show that the imidazolium‐modified electrode represents a solution for keeping the Rh model complex substantially selective and exhibiting a high energy efficiency for CO_2_ conversion to formic acid versus HER even with gas sources containing low concentrations of CO_2_ (10% v/v).

Furthermore, using a more complex CO_2_ stream corresponding to an incinerator simulated quasi‐flue gas, which contains diluted CO_2_ [1–10% mol] and impurities traces of SO_2_ [0–50 ppm] and NO_2_ [0–100 ppm],^[^ ^12^, ^19^ ^]^ we show that the modified electrode also limits the inhibition effect of NO_2_ and SO_2_ impurities during CO_2_RR. Here, we focus exclusively on NO_2_ and SO_2_ contaminants and do not include O_2_, as only a few examples of molecular catalysts based on Fe porphyrins or Co phthalocyanine have been shown, so far, to be capable to reduce CO_2_ in the presence of O_2_.^[^ ^42^, ^43^ ^]^ While some studies have analyzed the effect of traces of typical acidic impurities present in industrial incinerator flue gas, such as SO_2_ and NO_2_, on CO_2_RR catalyzed by solid electrocatalysts under 1 atm of CO_2_,^[^ ^44^, ^45^ ^]^ there is no comparable study using molecular catalysts. Such contaminants are likely to deteriorate the CO_2_RR performance since they might be prone to bind to and react with molecular catalysts^[^ ^46^ ^]^ and some of them are easier to reduce, at redox potentials required for CO_2_RR.^[^ ^47^ ^]^ We discuss here possible reasons for the limited effects of NO_2_ and SO_2_ impurities based on the role of the local electric field induced by imidazolium‐modified electrodes.

## 2 Results

**Figure 1:**
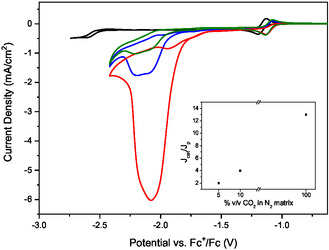
Cyclic voltammogram on a bare GC electrode of 2 mM the Rh complex and 0.5 M [TBA][PF_6_] either in CH_3_CN solution under argon (black plot) or in CH_3_CN solution containing 5% v/v H_2_O as a proton source under 100% CO_2_ (red plot), 10% v/v CO_2_ in a N_2_ matrix (blue plot), and 5% v/v CO_2_ in a N_2_ matrix (green plot). Inset: plot of catalytic current enhancement (*J*_cat_/*J*_p_) versus CO_2_ concentration. Scan rate: 0.01 V s^−1^.

**Figure** **1** shows the electrochemical characterization of the Rh complex by cyclic voltammetry (CV) on bare glassy carbon (GC) electrode (GCE) at a concentration of 2 mM in an acetonitrile solution containing 0.1 M [TBA][PF_6_] as a supporting electrolyte, under inert conditions (argon‐saturated electrolyte—black CV ), as well as in an acetonitrile solution with 5% v/v of H_2_O as a proton source in the presence of either pure (100% CO_2_—red CV) or diluted CO_2_ in a N_2_ matrix (5% and 10% v/v—green and blue CV, respectively).

The Rh complex represents a model molecular catalyst with a well‐established mechanism previously described in the literature.^[^ ^36^, ^38^ ^]^ The Rh complex under Ar (black plot in Figure 1) displays a reversible reduction signal at −1.19 V versus Fc^+^/Fc, corresponding to the two‐electron reduction of Rh(III) to Rh(I) coupled with the release of the chloro ligand and a second reduction wave corresponding to the one‐electron reduction of the bipyridine ligand,^[^ ^36^, ^38^ ^]^ which shifts from *E*_1/2_ = −2.53 V versus Fc^+^/Fc in the absence of CO_2_ and proton source to *E*_p_ = −2.07 V versus Fc^+^/Fc under catalytic conditions (in the presence of pure CO_2_% and 5% v/v H_2_O, red plot in Figure 1). This catalytic reduction wave is assigned to CO_2_RR and HER, as confirmed below via bulk electrolysis and in agreement with previous reports.^[^ ^38^, ^39^, ^40^ ^]^ In particular, the shoulder at *E*_p_ = −2.20 V versus Fc^+^/Fc is assigned to HER and the more anodic peak centered at −2.07 V to CO_2_RR. Figure 1 shows that upon dilution of CO_2_ (blue and green plots), the intensity of the CO_2_RR catalytic wave decreases: the *J*_cat_ */J*_p_ ratio, with *J*_cat_ being the maximum catalytic current density and *J*_p_ the peak current density under Ar, decreases from *J*_cat_ */J*_p_ = 13 for 100% CO_2_ to 4 for 10% CO_2_ and 2 for 5% CO_2_. Interestingly, *J*_cat_ */J*_p_ is not a linear function of CO_2_ concentration and catalysis remains significant even at 5% CO_2_.

**Figure 2:**
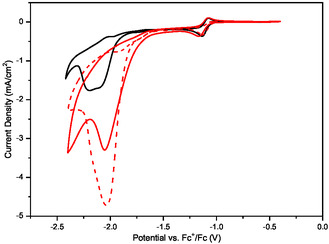
CV of 2 mM Rh complex and 0.5 M [TBA][PF_6_] in CH_3_CN solution containing 5% v/v H_2_O as a proton source under 100% v/v CO_2_ (dashed line) or 10% v/v CO_2_ in a N_2_ matrix (solid line) on bare GC electrode (black plot) and IM^+^_EE_‐modified electrode (red plots). Scan rate: 0.01 V s^−1^.

**Figure** **2** shows the electrochemical characterization of the Rh complex by CV under diluted CO_2_ (10% v/v) catalytic conditions on both a modified GCE (red solid plot) and a bare GCE (black solid plot), as well as under pure CO_2_ on the modified GCE (red dashed plot). In particular, the 1‐ethyl‐3‐methylimidazolium cation ([EMIM]^+^) was immobilized on the GCE following the previously described electrochemical procedure.^[^ ^37^ ^]^ The modified GCE is labeled as IM^+^_EE_‐modified electrode throughout this article. It contains a layer of positively charged imidazolium groups at the surface and is designed to enhance CO_2_RR and inhibit proton reduction by the Rh complex. The CV in Figure 2 reveals a remarkable effect of the modified electrode on the catalytic performance of the Rh complex under diluted CO_2_ (10% v/v), with a more intense and more anodic catalytic wave as compared to the catalytic wave on bare GCE. Indeed, a higher maximum catalytic current density (−3.3 mA cm^−2^, red solid plot) is observed on IM^+^_E_‐modified electrode in comparison with bare GCE (−1.9 mA cm^−2^, black solid plot), thus with a higher *J*_cat_ */J*_p_ ratio (*J*_cat_ */J*_p_ = 7). Remarkably, shifting from 100% to 10% CO_2_ results in only a modest decrease of the current density achieved on IM^+^_EE_‐modified electrode, as shown by comparing red dashed and solid plots in Figure 2. Furthermore, using 10% CO_2_, the catalytic onset is much more anodic (≈200 mV) on the IM^+^_EE_‐modified electrode than on bare GCE. Finally, the redox process associated with HER at *E*_p_ −2.20 V versus Fc^+^/Fc is slightly suppressed on the IM^+^_EE_‐modified electrode (red solid plot in Figure 2) compared to the bare electrode (black solid plot in Figure 2). These features are observed in all conditions using diluted CO_2_ as shown in Figure S1, Supporting Information (5% and 1% v/v CO_2_), where larger catalytic current densities and lower overpotentials are obtained on the IM^+^_EE_‐modified electrode. As shown in Figure S2, Supporting Information, reduction current associated with CO_2_RR in the absence of the Rh complex was only observed at very negative potentials, about 1 V more negative than in the presence of the Rh complex, on both bare and modified GCE, which demonstrates the key role of the Rh complex for CO_2_RR catalysis.

### 2.1 Bulk Electrolysis on Bare GC Electrode of Diluted CO_2_ in Batch Conditions

The catalytic performance of the Rh complex for CO_2_RR on bare GCE under diluted CO_2_ (10% v/v CO_2_) was evaluated by bulk electrolysis at constant current density (controlled current electrolysis [CCE] −1.2 mA cm^−2^) during 3.5 h. This current density corresponds to a lower absolute value than the peak current density (−1.9 mA cm^−2^) displayed in Figure 2 and ensures high selectivity toward CO_2_RR during electrolysis. The outcome from CCE under 10% CO_2_ was compared to the one obtained under 100% CO_2_. In both cases, formate (HCOO^−^) was the sole product detected in the liquid phase by ionic chromatography analysis and hydrogen (H_2_) the only gas product detected by gas chromatography. First, batch conditions were used with electrolytes only saturated with the corresponding CO_2_ stream at the beginning of the electrolysis. **Table** **1** summarizes the faradaic efficiencies obtained for HCOO^−^ (FE_HCOO_–) and H_2_ (FE_H2_) as a function of the concentration of the CO_2_ gas stream used in the electrolysis. The mass balance of the electrolysis is efficiently closed and reaches a total FE (FE_HCOO_– + FE_H2_) near 90% in both cases.

**Table 1:** CCE on a bare GCE at −1.2 mA cm^−2^ of 2 mM the Rh complex and 0.5 M [TBA][PF_6_] in CH_3_CN solution containing 5% v/v H_2_O and different CO_2_ streams in a two compartments H‐type cell under batch conditions. Total circulated charge 15 C. Solution stirring rate: 300 rpm.

CO_2_ v/v [%]	*E*_cat_^average^ [V vs Fc^+^/Fc]	FE_HCOO_ *–* a) [%]	FE_H2_ a) [%]
100	−1.65	58 ± 4	29 ± 4
10	−2.19	48 ± 3	38 ± 4

a) Faradaic efficiencies after a passed total charge of 15 C.

**Figure 3:**
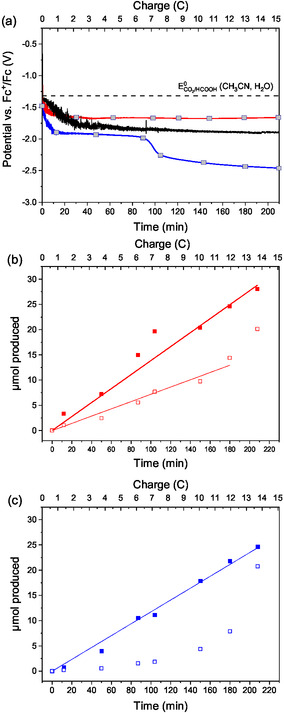
a) Bare GC cathode potential evolution during CCE in H‐type cell at −1.2 mA cm^−2^ of 2 mM of the Rh complex and 0.5 M [TBA][PF_6_] in CH_3_CN solution containing 5% v/v H_2_O under different gas saturation conditions: saturated atmosphere of 100% CO_2_ (red plot), 10% v/v CO_2_ in a N_2_ matrix (blue plot), or under continuous gas flow (14 mL min^−1^) of 10% v/v CO_2_ in a N_2_ matrix (black plot). Solution stirring rate 300 rpm. b) Production of H_2_ (empty squares) and HCOO^−^ (filled squares) for saturated atmosphere of 100% CO_2_ and c) saturated atmosphere of 10% CO_2_ in a N_2_ matrix. Linear regression lines are also included. H_2_ and HCOO^−^ quantification samples are indicated by gray squares in graph (a).

**Figure** **3**a shows the cathode potential response as a function of electrolysis time, which is compared to the thermodynamic potential of CO_2_ to HCOOH under the studied experimental conditions. The cathode potential evolution under 100% CO_2_ saturation (red plot) is perfectly stable all along CCE. In contrast, the cathode potential evolution under 10% v/v CO_2_ saturation (blue plot) exhibits a more negative value, as well as a sudden decrease after 90 min of electrolysis followed by a second plateau at a more cathodic potential. In contrast, no cathode potential drop is observed when the 10% v/v CO_2_ gas stream is continuously flowing in the electrolyte all along the electrolysis (black plot). Thus, the potential drop under batch conditions using diluted CO_2_ is likely due to a shortage in CO_2_ after 90 min electrolysis, not occurring with 100% CO_2_, leading to the shifting in the main reaction catalyzed by the Rh complex from CO_2_RR to HER in order to cope with the imposed current density. Figure 3b,C supports this interpretation. They report the time course of formate and H_2_ production as a function of electrolysis time using 100% CO_2_ or 10% v/v CO_2_ under batch conditions, respectively. The production of formate was approximately linear with time all along the electrolysis in both cases (filled squares in Figure 3b,c, *R* ^2^ = 0.9931 and 0.9959, respectively) with a rate slightly lower at 10% CO_2_ as compared to 100% CO_2_. In contrast, the evolution of H_2_ production (empty squares in Figure 3b,c) was different depending whether CO_2_ was pure (100%) or diluted (10%). Figure 3b (100% CO_2_) shows a quasi‐linear time dependence of H_2_ production (*R* ^2^ = 0.9849), whereas Figure 3c (10% CO_2_) shows a greater acceleration of H_2_ production after a low production during the first 100 min of electrolysis. Interestingly, the initial H_2_ production rate is lower at 10% CO_2_ as compared to that at 100% CO_2_, so that selectivity for CO_2_RR, as probed by the formate/H_2_ ratio, is larger using diluted CO_2_ (formate/H_2_ = 4) as compared to pure CO_2_ (formate/H_2_ = 2) during the first 100 min of electrolysis.

### 2.2 Bulk Electrolysis of Diluted CO_2_ in Flow Conditions

**Figure 4:**
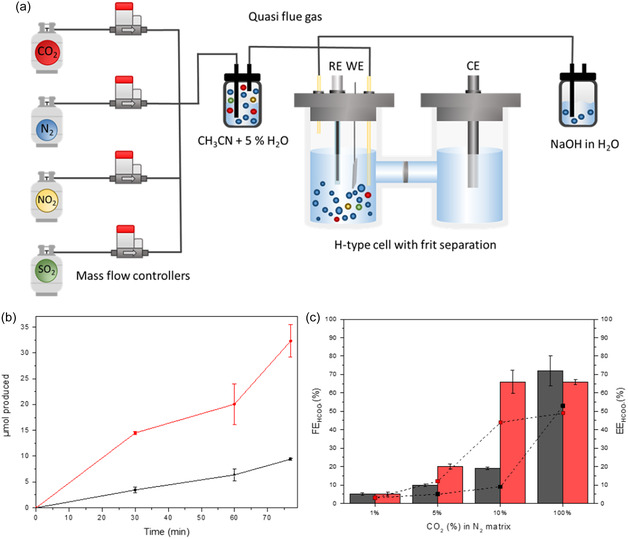
a) Schematic illustration of the bulk electrolysis setup in a two compartments H‐type cell under flow conditions for diluted CO_2_ and simulated quasi‐flue gas experiments, highlighting the placement of a gas humidifier at the gas inlet and a gas trap at the gas outlet, which ensures optimal gas hydration and capture of exhaust gases. The outlet of the gas trap is open to the atmosphere to prevent overpressure due to continuous gas flow during CCE experiments. Mass flow controllers regulate the flow of pure gases and custom‐made bottles containing CO_2_, SO_2_, and NO_2_ (each diluted in N_2_), allowing precise gas mixture preparation for studies. b) Formate production during CCE at −3.3 mA cm^−2^ of 2 mM the Rh complex and 0.5 M [TBA][PF_6_] in CH_3_CN solution containing 5% v/v H_2_O and under a continuous gas flow of 14 mL min^−1^ of 10% v/v CO_2_ in a N_2_ matrix on the bare GC electrode (black plot) and the IM^+^_EE_‐modified electrode (red plot). c) FE_HCOO_ *–* (bars) and EE_HCOO_ *–* (symbols) after 15 C of circulated charge from different CCE at −3.3 mA cm^−2^ on bare GC electrode (black) and IM^+^_EE_‐modified electrode (red) as a function of the type of dilution of CO_2_ in a N_2_ matrix (1% v/v CO_2_, 5% v/v CO_2_, 10% v/v CO_2_, or 100% v/v CO_2_).

The catalytic performance of the Rh complex for CO_2_RR using a bare GC and an IM^+^_EE_‐modified electrode under 100% CO_2_ and diluted CO_2_ (10%, 5% and 1% v/v CO_2_) was evaluated by bulk CCE during 76 min at −3.3 mA cm^−2^ (**Table** **2**). In all cases, the CO_2_ stream was continuously flowed in the electrolyte all along the electrolysis, as schematically described in **Figure** **4**a, to avoid any shortage in CO_2_ substrate and subsequent drop in the cathode potential. Note that under flow configuration, the H_2_ produced was not quantified and thus total FE ≤ 72% was reached in all cases since only formate production was reported. Nevertheless, it can be assumed that the missing circulated charge corresponded to H_2_ production based on the previous results obtained under batch conditions and reported in Table 1, where formate and H_2_ were the only detected products. Figure 4b nicely shows a much faster (3.5 fold) formate production rate using the IM^+^_EE_‐modified cathode (red plot) as compared to the bare GCE (black plot) during electrolysis of diluted 10% v/v CO_2_, reflecting again the positive effect in CO_2_RR of the modified electrode. Figure 4c compares the Faradaic efficiency values for formate (FE_HCOO_ *–*) obtained at the end of the electrolysis using the IM^+^_EE_‐modified electrode (red bars) to those obtained with the bare GCE (black bars) when flowing different types of CO_2_ streams (100%, 10%, 5%, and 1% v/v CO_2_). As expected, a dramatic decrease of FE_HCOO_ *–* in both electrodes is observed as the result of decreased concentration of available CO_2_, from a pure CO_2_ to 1% CO_2_ stream. Interestingly, this decrease is much more limited for the imidazolium‐modified electrode. This is particularly visible using 10% CO_2_ (red bars), where the FE_HCOO_ *–* remains constant at 66% as for 100% CO_2_, as compared to 19% with GCE (black bars), and to a lesser extent at 5% CO_2_. Thus, the imidazolium‐modified electrode allows to keep an energy efficiency for formate production (*EE*_HCOO_ *–*) close to 50% using a diluted 10% v/v CO_2_ stream. Table 2 summarizes the results from all bulk CCE at −3.3 mA cm^−2^ for both diluted CO_2_ and simulated quasi‐flue gas streams under flowing conditions, including the final formate concentration [HCOO^−^] and cathode potential (*E*), as well as cathodic overpotential (*η*), FE_HCOO_ *–* and EE_HCOO_–.

**Table 2:** CCE at −3.3 mA cm^−2^ of 2 mM the Rh complex and 0.5 M [TBA][PF_6_] in CH_3_CN solution containing 5% v/v H_2_O in a two compartments H‐type cell under continuous purging flow (14 mL min^−1^) of different CO_2_ streams without or with impurities NO_2_ [100–400 ppm] or SO_2_ [50–200 ppm] in bare GC electrode or IM^+^_EE_‐modified electrode. Two control experiments with no Rh complex are also included. Total circulated charge 15 C. Solution stirring rate: 300 rpm.

CO_2_ v/v [%]	Gas impurity [ppm]	IM^+^_EE_‐modified electrode	[HCOO^−^] [μmol/L]^a^^)^	*η* [V]^b^^)^	*E* [V] versus Fc^+^/Fc^c^^)^	FE_HCOO_ *–* [%]^d^^)^	EE_HCOO_ *–*. [%]^e^^)^
100^f^^)^	–	No	587 ± 163	2.00	−3.32	14 ± 4	6 ± 1
100^f^^)^	–	Yes	446 ± 54	1.98	−3.30	11 ± 1	4 ± 1
100	–	No	2844 ± 356	0.44	−1.76	72 ± 9	54 ± 7
100	–	Yes	2608 ± 11	0.44	−1.76	66 ± 1	50 ± 1
10	–	No	756 ± 17	1.75	−3.07	19 ± 1	8 ± 1
10	–	Yes	2599 ± 252	0.70	−2.02	66 ± 6	43 ± 4
5	–	No	401 ± 14	1.50	−2.82	10 ± 1	4 ± 1
5	–	Yes	796 ± 45	0.79	−2.11	20 ± 1	13 ± 1
1	–	No	214 ± 16	1.10	−2.42	5 ± 1	3 ± 1
1	–	Yes	185 ± 10	0.79	−2.11	5 ± 1	3 ± 1
10	NO_2_, 100	No	460 ± 51	1.86	−3.18	12 ± 1	5 ± 1
10	NO_2_, 100	Yes	1125 ± 127	0.90	−2.22	29 ± 4	17 ± 2
10	NO_2_, 200	Yes	1432 ± 105	0.80	−2.11	35 ± 5	22 ± 3
10	NO_2_, 400	Yes	821 ± 171	0.82	−2.14	21 ± 5	13 ± 3
10	SO_2_, 50	No	560 ± 177	1.95	−3.27	14 ± 4	6 ± 2
10	SO_2_, 50	Yes	1317 ± 252	0.89	−2.21	33 ± 7	18 ± 2
10	SO_2_, 100	Yes	1692 ± 152	1.03	−2.32	42 ± 5	20 ± 3
10	SO_2_, 200	Yes	1208 ± 88	0.80	−2.11	31 ± 2	13 ± 3

a) Total formate concentration at the end of the CCE (total volume of electrolyte 12.5 mL).

b) Cathodic overpotential calculated at final electrolysis time.

c) Cathodic potential at the end of the CCE.

d) Faradaic efficiency for formate (FE_HCOO_ *–*) after a total circulated charge of 15 C.

e) Energy efficiency for formate (EE_HCOO_ *–*) after a total circulated charge of 15 C.

f) Control CCEs performed in the absence of the Rh catalyst.

For the case of diluted 10% v/v CO_2_ stream, all parameters reported in Table 2 reflect a higher performance with the IM^+^_EE_‐modified electrode than GCE. Besides larger formate selectivity, probed by FE_HCOO_ *–* increasing from 19% to 66%, already discussed above, we observe a large decrease of the cathode potential by more than 1 V (translated into a 1.05 V decrease of the cathodic overpotential *η*—see Figure S3b, Supporting Information) and thus a fivefold increase of the EE_HCOO_ *–.* However, while the modified electrode system is still significantly superior to the bare electrode one using 5% CO_2_ gas stream, this is not the case anymore for a too diluted CO_2_ stream (1% CO_2_). Figure S3, Supporting Information, shows not only a more anodic cathode potential during electrolysis with the IM^+^_EE_‐modified cathode (red plots) as compared to the bare GC cathode (black plots) but also a more stable potential behavior for all three diluted CO_2_ streams. Moreover, additional blank electrolysis at −3.3 mA cm^−2^ were performed in order to exclude the potential contribution in CO_2_RR activity from Rh^0^ nanoparticles deposited on the GCE due to the decomposition of the Rh molecular catalyst during electrolysis. Tables S1–S2, Supporting Information, show the results of two consecutive electrolysis performed on either the same bare GCE or the IM^+^_EE_‐modified electrode, respectively. On the first electrolysis under catalytic conditions, formate is the main product on both electrodes. In contrast, on the second electrolysis, using the same electrode after a rinsing step, but in the absence of the Rh complex in solution, a minor production of formate and FE_H2_ larger than 80% are detected. This excludes deposited Rh nanoparticles as the catalytic species for CO_2_ conversion to formate.

### 2.3 Bulk Electrolysis of Simulated Quasi‐Flue Gas in Flow Conditions

**Figure 5:**
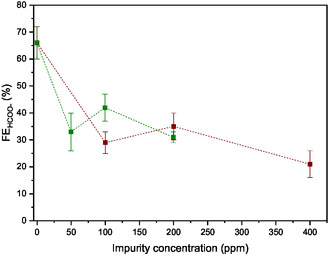
FE_HCOO_ *–* after 15 C of circulated charge from different CCE at −3.3 mA cm^−2^ of 2 mM Rh complex and 0.5 M [TBA][PF_6_] in CH_3_CN solution containing 5% v/v H_2_O on IM^+^_EE_‐modified electrode as a function of the amount of either NO_2_ (brown squares) or SO_2_ (green squares) present in diluted 10% v/v CO_2_ in N_2_ matrix (continuous purging flow of 14 mL min^−1^).

Next, we explore the effect on CO_2_RR activity and selectivity of the presence of gas impurities, specifically NO_2_ and SO_2_ in a diluted CO_2_ stream (10% v/v CO_2_ in N_2_ matrix), and the possible advantage of using the IM^+^_EE_‐modified cathode. Thus, these impurities were introduced separately in the diluted CO_2_ gas stream at trace concentrations simulating those found in industrial incinerator flue gas^[^ ^12^, ^19^ ^]^ (SO_2_ at 50 ppm and NO_2_ at 100 ppm), as schematically described in Figure 4a. Figure S4, Supporting Information, reports the cathode potential versus time evolution during CCE at −3.3 mA cm^−2^ comparing the bare GCE (black plots) and the IM^+^_EE_‐modified electrode (red plots) in a diluted 10% CO_2_ stream without (solid line) and with NO_2_ (dashed line) or SO_2_ (dotted line) during 76 min of electrolysis. It shows almost no effect of impurities on cathode potential, which allows keeping a significant overpotential decrease on IM^+^_EE_‐modified electrode versus GCE of 0.96 V and 1.06 V (see Table 2) in the presence of NO_2_ and SO_2_, respectively. Table 2 demonstrates the deleterious effect of both NO_2_ and SO_2_, even at such low concentrations, on formate production on both electrodes. Nevertheless, Table 2 also shows the positive effect of the modified electrode regarding the concentration of formate at the end of CCE, FE_HCOO_ *–* and EE_HCOO_ *–* values, which are more than twice greater working with the IM^+^_EE_‐modified cathode as compared to the bare GCE: compare FE_HCOO_ *–* = 29% (NO_2_) and 33% (SO_2_) on the IM^+^_EE_‐modified cathode to FE_HCOO_ *–* = 12% (NO_2_) and 14% (SO_2_) on the bare GCE. Thus, CO_2_RR occurs at more anodic potentials and is more selective to formate production on the IM^+^_EE_‐modified electrode, even in the presence of either SO_2_ or NO_2_ impurities. **Figure** **5** shows the effect of increasing the concentration of those impurities, either NO_2_ [100–400 ppm] or SO_2_ [50–200 ppm], in the diluted CO_2_ stream (10% v/v CO_2_ in N_2_ matrix). A very limited impact on FE_HCOO_ *–* is observed in both cases, exhibiting a minor decrease from 29% to 20% in the presence of 400 ppm NO_2_ and from 33% to 30% in the presence of 200 ppm SO_2_. Figure S5, Supporting Information, reports the cathode potential versus time evolution during those electrolysis on the IM^+^_EE_‐modified electrode. Figure S6, Supporting Information, displays the linear sweep voltammetry profiles corresponding to the Rh complex in solution together with H_2_O as proton source, 5% v/v NO_2_, 5% v/v SO_2_, or 5% v/v CO_2_ on bare and modified GCE to evaluate its affinity to react with each of those compounds.

## 3 Discussion

Achieving CO_2_RR using a gas stream released from industrial plants directly, without CO_2_ purification, is a great challenge. This is largely related to the competing reactions occurring specifically when the CO_2_ substrate accounts for a small part of the gas, making CO_2_RR more kinetically unfavorable. Among these competing reactions, HER is the most prominent one, but catalytic reduction of gas impurities such as NO_2_ and SO_2_ by molecular complexes can also deteriorate, even at the low concentration found in some industrial flue gases, the CO_2_RR performance during electrolysis because of the similar redox potentials required for CO_2_, NO_2_, and SO_2_ reductions.^[^ ^44^, ^47^ ^]^ Our results provide an original illustration that not only a molecular catalyst, such as the model Rh complex used, allows significant CO_2_RR activity when CO_2_ accounts only for 5% and 10% of the gas mixture, in line with rare previous examples of other molecular catalysts converting diluted CO_2_ streams,^[^ ^25^, ^26^, ^27^ ^]^ but, more importantly, that this is possible thanks to a molecular modification on the GCE. Here, the electrode is modified with a positively charged imidazolium layer providing a suitable local environment, which limits HER contribution and enhances CO_2_RR performance when the gas stream contains little CO_2_ (5% and 10%), as well as redox active impurities (NO_2_ or SO_2_), an observation with no precedent in molecular catalysis literature.

As previously established experimentally and computationally by DFT calculations in homogeneous systems,^[^ ^36^ ^]^ we propose that the effect of the imidazolium‐based modified electrode is due to the ability of the Rh complex to develop π–π interactions with the imidazolium group on the electrode surface, even though such interactions might be weaker in the case of immobilized imidazolium as compared to imidazolium in solution. First, this would indeed make redox potentials of the catalyst more positives and facilitate its reduction to generate the catalytically active intermediate, which explains the observed decrease in cathodic overpotential, as well as in full cell potential. Second, it contributes to inhibit HER via electrostatic repulsion between the positively charged imidazolium and free protons and via increasing the free‐energy barrier of the HER pathway, which explains the enhanced selectivity observed for formate production. Third, it would help accumulating the active form of the molecular catalyst at the surface of the electrode, explaining the increased current density achieved. Such a concentration effect would partly compensate for the reduction of CO_2_ concentration available under diluted gas streams. Finally, in agreement with previous reports describing strong interactions between CO_2_ and imidazolium^[^ ^48^, ^49^ ^]^ the surface modification might also contribute to concentrate CO_2_ at the electrode interface. All these effects are strong enough to offset the low CO_2_RR kinetics associated with the low concentration of CO_2_ in the gas stream.

In conclusion, there is a clear negative effect of adding traces of NO_2_ (100–400 ppm) and SO_2_ (50–200 ppm) on CO_2_RR catalyzed by the Rh complex. Our working hypothesis for this behavior is that the same Rh‐hydride active catalytic intermediate might react with NO_2_ and SO_2_, in competition with CO_2_. Figure S6, Supporting Information, shows a comparative study under identical concentration (5% v/v in N_2_) of individually added NO_2_, SO_2_, and CO_2_ in the presence of the Rh complex and either on bare GCE or IM^+^_EE_‐modified electrode. Figure S6a, Supporting Information, on bare GCE exhibits a nonzero reduction current associated with the presence of both NO_2_ and SO_2_ within the same potential window where the Rh complex catalyzes CO_2_RR (*E* < −2.0 V vs Fc^+^/Fc), being CO_2_ reduction the main contribution up to −2.25 V, from where the reaction associated to NO_2_ becomes the most relevant current contributor. In contrast, Figure S6b, Supporting Information, on IM^+^_EE_‐modified electrode exhibits CO_2_ reduction as the main current contribution up to −2.40 V, despite nonzero reduction current associated with the presence of both NO_2_ and SO_2_ is also displayed within the same potential window. These voltammograms indicate that both NO_2_ and SO_2_ present some affinity for the Rh complex and compete for its active form. Notably, the IM^+^_EE_‐modified electrode exhibits, in addition to the selective enhancement of CO_2_RR in comparison with bare GCE, a moderate inhibition effect for the reaction associated to the NO_2_ presence, because the catalytic current in the presence of NO_2_ is half of that exhibited on bare GCE at −2.40 V. However, further studies are required for reaching a deep mechanistic understanding of all those associated processes, which are out of the scope of the present work. Interestingly, the modified electrode surface provides a significant decrease in cathodic overpotential and helps maintaining a reasonable selectivity for formate production, allowing a FE_HCOO_ *–* value more than twofold larger as compared to a bare electrode (Table 2). Thus, for example with challenging quasi‐flue gas streams containing 10% CO_2_ and 50 ppm SO_2_ in a N_2_ matrix, a FE_HCOO_ *–* value of 33% and EE_HCOO_ *–* of 20% were obtained. As the matter of fact, the negative effect of adding traces of NO_2_ and SO_2_ in the diluted CO_2_ stream seems to be concentration independent below 400 ppm. Furthermore, the enhanced CO_2_RR performance exhibited on the modified electrode in the presence of NO_2_ and SO_2_ impurities in comparison with bare GCE might be linked to the different dipolar moments displayed by all three competing reactants and its orientation within the electrode/electrolyte interface due to the local electric field effect: CO_2_ (linear molecule, zero dipolar moment, insensitive to electric field effect) and NO_2_ and SO_2_ impurities (bent molecules, nonzero dipolar moment, sensitive to electric field effect). Thus, the modified electrode, thanks to the local electrostatic effect induced by the positively charged imidazolium, is likely to impact on the molecular orientation of NO_2_ and SO_2_, which might reduce the probability to reach a strong interaction with the Rh complex and thus, difficult the competitive NO_2_ and SO_2_ reduction reactions.

## 4 Conclusion

In conclusion, our results demonstrate the potential of appropriate surface modifications on noncatalytic GC electrodes for achieving CO_2_RR catalyzed by molecular catalysts using simulated gas streams from industrial plants, containing low share of CO_2_ and deleterious gaseous impurities. This is illustrated here with a model molecular Rh complex allowing high selectivity for formate production from gas mixtures containing 5–10% CO_2_ and traces of NO_2_ and SO_2_, thanks to the presence of a positively charged imidazolium layer on the cathode of the electrolyzer. These results point out surface modification of the electrode as an interesting approach combined with molecular electrocatalysts to treat direct industrial CO_2_ streams and overcome the expenses associated with CO_2_ capture, separation, and subsequent purification.

Finally, the choice of a water soluble molecular catalyst as the Rh complex, together with applying a high constant current during electrolysis, properly places the results of the present work as the initial step toward large‐scale applications. Further work devoted to evaluate the feasibility of formate production from quasi‐flue gas in acidic aqueous solution in a flow cell‐type reactor is required for that purpose.

## 5 Experimental Section

5.1

5.1.1

##### Reactants

Anhydrous acetonitrile of 99.99% purity (CH_3_CN), tetrabutyl ammonium hexafluorophosphate ([TBA][PF_6_], >99%) 2,2′‐Bipyridyl of ≥99% purity, and ferrocene (98%) were purchased from Sigma–Aldrich. The Rhodium complex precursor dichloro(pentamethylcyclopentadienyl) rhodium(III) dimer [Rh(Cp*)Cl_2_]_2_ of 99% purity was purchased from Strem Chemicals or from Merck (Chiralyst P618). The ionic liquid 1‐ethyl‐3‐methylimidazolium hexafluorophosphate ([EMIM][PF_6_]) (99%) was acquired from Io‐Li‐Tec (Germany). All reactants were utilized without any additional purification. Ultrapure water (18.2 MΩ cm, Millipore) was used to conduct experiments.

##### Synthesis of Rhodium Complex

The described synthesis of [Rh(bpy)(Cp*)Cl]Cl was previously adapted within the research team, from protocols outlined in the existing literature.^[^ ^50^ ^]^ An ethanol solution (30 mL) of 1 equivalent [Rh(Cp*)Cl_2_]_2_ (200 mg, 0.32 mmol) and 2 equivalents 2,2′‐bipyridine (120 mg, 0.76 mmol) was stirred at room temperature for 2 h in the dark. Evaporation to dryness of the resultant solution, clear and orange‐yellow in appearance, was performed next. The yellow solid was dissolved in a minimal quantity of acetonitrile (CH_3_CN) and subsequently precipitated by adding ethyl acetate (AcOEt). The precipitate was then collected on a Buchner funnel and dried under vacuum. The dried powder purity was verified by ^1^H NMR spectroscopy according to the literature. The ^1^H NMR spectrum of [Rh(bpy)(Cp*)Cl] is shown in Figure S7, Supporting Information (300 MHz, CD_3_CN): δ/ppm, 1.66 (s, 15H), 7.88 (dt, *J* = 6.5 Hz, 2H), 8.34 (dt, *J* = 7.9 Hz, 2H), 8.70 (d, *J* = 7.9 Hz, 2H), 8.99 (d, *J* = 5.4 Hz, 2H).

##### Electrochemical Studies

CV and chronopotentiometry experiments were performed on Biologic Science Instruments SAS potentiostats (SP‐300 or VSP‐300). All electrochemical trials were conducted at room temperature (20 ± 2 °C) in acetonitrile (CH_3_CN) with 5% vol. H_2_O content. [TBA][PF_6_] was used as a supporting electrolyte in solution (0.5 M). Pure gases Ar (>99.99%), N_2_ (>99.99%), and CO_2_ (>99.99%) used to saturate solutions were purchased from Air Liquide. Bottles containing the diluted gases: 10% v/v CO_2_ in a N_2_ matrix, 5% v/v NO_2_ in a N_2_ matrix, and 5% v/v SO_2_ in a N_2_ matrix were custom made by Air Liquide. The electrochemical studies under 10% v/v CO_2_ in N_2_ matrix atmosphere were conducted using the custom‐made gas bottle from Air Liquide to saturate the solution. For lower CO_2_ concentrations (5% v/v and 1% v/v CO_2_ in a N_2_ matrix), the gas mixtures used to purge the solutions were prepared by properly mixing pure CO_2_ and N_2_ gases at different ratios using mass flow controllers (Bronkhorst, EL‐FLOW Prestige, model FG‐201CV). Gas flow rates were controlled online using FlowSuite 2 software. The preparation of the simulated quasi‐flue gas was done by simultaneously mixing pure CO_2_ and N_2_ gases, as well as SO_2_ and NO_2_ (Bronkhorst's mass flow controllers, EL‐FLOW Prestige, model FG‐200CV) from the custom‐made bottles containing 5% v/v SO_2_% and 5% v/v NO_2_ in a N_2_ matrix, respectively. A three‐electrode setup was used to carry out CV experiments, using a GC disc (diameter = 3 mm, area = 0.07 cm^2^, BioLogic) as working electrode (WE), a platinum wire (diameter = 0.5 mm, Alfa Aesar, 99.5% purity) as a counter electrode (CE), and an Ag/AgCl/KClsat reference electrode (RE). The WE was polished on a polishing cloth (DP‐Nap 200 mm, Struers) on a 1 μm diamond suspension (Struers), followed by a 10 s sonication in water and subsequent drying before initiating experiments. The CE was previously flame annealed and the RE was separated from the solution in all cases using a salt bridge. All potentials were calibrated at the end of each experiment by adding the ferrocenium/ferrocene (Fc^+^/Fc) redox couple as an internal standard, since they were carried out in CH_3_CN. CVs were run at 0.01 V s^−1^ scan rate and only the third steady‐state cycle of all CVs is shown. *E*_1/2_ potential (V) corresponds to the half wave potential from CV. *E*_p_ potential (V) corresponds to the potential at the peak current density. Catalytic response from CV was determined by calculating the current density ratio (*J*_cat_ */J*_p_), where (*J*_cat_) corresponds to the catalytic current density in the presence of CO_2_ and H_2_O; meanwhile, (*J*_p_) corresponds to the reduction peak current density under inert conditions (Ar).

##### Modified Electrode Synthesis by Imidazolium Immobilization on GCEs (IM^+^_EE_‐Modified Electrode)

The grafting of imidazolium (IM^+^) was done by an electrochemical−electrochemical (EE) two‐step covalent method, optimized in our prior study,^[^ ^37^ ^]^ and gives a result an IM^+^_EE_‐modified electrode. The IM^+^ immobilization was carried using a three‐electrode cell: a GC disc or plate (Alfa Aesar) as a WE, where the grafting took place, a GC rod as a CE, and an all‐solid Ag/AgCl wire directly immersed in solution was used as the pseudoreference electrode. The IM^+^ immobilization occurs in a 0.5 M [EMIM][PF_6_] in CH_3_CN solution purged with argon by using CV (scan rate 0.1 V s^−1^) as the electrochemical deposition technique. It involves two reaction steps: (1) the electrochemical reduction of [EMIM]^+^ on the GC electrode by performing a forward sweep from −1.45 to −3.95 V versus Fc^+^/Fc and (2) a subsequent electrochemical oxidation step in the reverse scan of CV at open‐circuit potential to form the positively charged imidazolium layer on the GC electrode. After this, the modified electrode (IM^+^_EE_) was removed from the cell, washed and sonicated 10 s in acetonitrile, and placed in a new solution for further analysis and evaluation of its catalytic properties.

##### CO_2_ Conversion by Bulk Electrolysis

A gastight two‐compartment electrochemical H‐type glass cell with a glass frit separating anolyte (3 mL) and catholyte (9.5 mL) solutions was used in all electrolysis reported here. CCE was performed at different current density values in acetonitrile solution containing 5% v/v H_2_O and 0.5 M of supporting electrolyte ([TBA][PF_6_]) previously saturated by gas bubbling (100%–1% CO_2_ or the different quasi‐flue gas mixtures) in both anolyte and catholyte, during 5 and 30 min, respectively. The gas was humidified in a solution containing acetonitrile +5% v/v H_2_O before entering the H‐cell compartments. For experiment under static conditions, no continuous CO_2_ gas was purged during the electrolysis; meanwhile, experiments under continuous flow conditions were purged in the catholyte with CO_2_ gas, diluted gas, or quasi‐flue gas mixture at a flow rate of 14 mL min^−1^. 2 mM of the Rh complex was only added in the catholyte. The working electrode was a 1 cm^2^ GC plate (1 mm thick, type 2, from Alfa Aesar), the counter electrode was a 5 cm^2^ GC rod (Alfa Aesar), and a conventional Ag/AgCl/KClsat electrode separated from the solution by a salt bridge, which was calibrated with ferrocene as an internal redox reference, was used as a reference electrode. Ohmic losses in the cell were minimized by achieving the minimal distance between electrodes and keeping magnetic stirring during the electrolysis. In all cases, the current density was calculated using the electrode geometrical area. All CCE experiments were performed with two or three replicates to check results reproducibility.

##### Diluted CO_2_ Gas and Simulated Incinerator Quasi‐Flue Gas

CCE experiments under diluted (10%–1% CO_2_) or the different quasi‐flue gas mixtures were performed by purging both anolyte and catholyte compartments. A flow rate of 14 mL min^−1^ was used to purge the electrochemical cell under continuous flow conditions. Studied impurities concentration range was either (50–200) ppm of SO_2_ or (100–400) ppm of NO_2_ in 10% v/v CO_2_ diluted in a N_2_ matrix. An additional gas trap open to the atmosphere containing 0.1 M NaOH was added at the outlet of the H‐cell compartments when studying the effects of impurities to avoid the release of these gases in the open atmosphere.

##### Analytical Quantification of Products

Formate (HCOO^−^) was the sole product detected in the liquid phase by ionic chromatography analysis (Figure S8, Supporting Information) and hydrogen (H_2_) was the only gas product detected by gas chromatography from CO_2_RR bulk CCE. H_2_ was quantified by gas chromatography (Model 8610C, SRI Instruments) equipped with a TCD detector and any other potential gas product was evaluated by the FID detector from 100 μL gas aliquots of the headspace of both compartments in the H type cell. The GC was periodically calibrated using a custom standard gas mixture in CO_2_ (0–85 nmol H_2_) (Figure S9a, Supporting Information). Formate was evaluated using an ionic exchange chromatograph (IC) (Metrohm 883 Basic IC), automatized by a 863 Compact IC autosampler and equipped with a Metrosep A Supp 5 column and a conductivity detector. A solution of 1 mM NaHCO_3_ and 3.4 mM Na_2_CO_3_ was used as eluent with a flow of 0.7 mL min^−1^. Calibration curve for HCOOH quantification (0–15 μM) was periodically checked before analysis (Figure S9b, Supporting Information). Formate samples for IC quantification were prepared by diluting 200 times in ultrapure water a sample of 50 μL from both the catholyte and anolyte solutions. 20 μL of the diluted sample were injected into the IC chromatograph.

Faraday efficiency (FE, %) of each reaction product is calculated from the ratio between the charge consumed to form each product and the total circulated charge.^[^ ^51^ ^]^ A constant total circulated charge (15 C) was used in all electrolysis in order to reach a fair comparison among all CCE results. However, this total circulated charge is corrected to account for the initial three electrons consumed by the Rh complex (2 mM in solution) to generate its active form. Catalyst activation charge = [number of electrons × Faraday constant × mol of catalyst] = [3 × 96485 × 1.9 × 10^−5^] = 5.47 C and thus effective circulated charge is 9.5 C in all bulk electrolysis. The cathodic overpotential (*η*) was calculated from the difference between the cathode potential at final electrolysis time and *E* ^0^_CO2/HCOOH_ (CH_3_CN, H_2_O) = −1.32 V versus Fc^+^/Fc in acetonitrile.^[^ ^52^ ^]^ The cathodic half reaction energy efficiency (EE, %) was calculated for CO_2_ conversion to formate reaction according to the following Equation (1).^[^ ^53^ ^]^

(1) $$\text{Cathodic energy efficiency, EE}\left(\%\right)\;=\;\left(E_{T}/E\right)\;\times {\text{FE}}_{\text{HCOO}-}$$

where *E*_T_ is the thermodynamic potential in volts required for the electrocatalytic reduction of CO_2_ to formate (*E* ^0^_CO2/HCOOH_ (CH_3_CN, H_2_O) = −1.32 V vs Fc^+^/Fc), whereas *E* and FE_HCOO−_ represent the experimental obtained cathode potential in volts (vs Fc^+^/Fc) and the formate Faradaic efficiency (%), respectively.

## Conflict of Interest

The authors declare no conflict of interest.

## Supporting information

Supplementary Material

## Data Availability

The data that support the findings of this study are available from the corresponding author upon reasonable request.
