# Supplementary material for: Formate Production from Simulated Quasi‐Flue Gas Combining a Molecular Catalyst and a Modified Electrode
Source: ChemSusChem. 2025 Jun 13;18(14):e202500392. doi: 10.1002/cssc.202500392 (PMC12270365; doi:10.1002/cssc.202500392)
Supplement: Supplementary file 1 — Supplementary Material [file CSSC-18-e202500392-s001.pdf]

# SUPPORTING INFORMATION

## Formate Production from Simulated Quasi Flue Gas Combining a Molecular Catalyst and a Modified Electrode

*Yutzil Segura-Ramirez[a,b], Maria Gomez-Mingot[a,*], Marc Fontecave[a,*], Carlos M. Sánchez-Sánchez[b,*]*

[a] Laboratoire de Chimie des Processus Biologiques, Collège de France, UMR 8229 CNRS, Sorbonne Université, PSL Research University, 11 Place Marcelin Berthelot, 75005 Paris, France

[b] Sorbonne Université, CNRS, Laboratoire Interfaces et Systèmes Electrochimiques (LISE), 4 Place Jussieu, 75005 Paris, France

**Corresponding authors e-mail addresses:**

* maria.gomez@college-de-france.fr
* marc.fontecave@college-de-france.fr
* carlos.sanchez@sorbonne-universite.fr

## Table of Content

**Figure S1.** Cyclic voltammograms of 2 mM Rh complex and 0.5 M [TBA][$PF_6$] in $CH_3CN$ solution, all containing 5% v/v $H_2O$ on bare GC and $IM^+_{EE}$ modified electrode under (a) 5% v/v $CO_2$ and (b) 1% v/v $CO_2$ in a $N_2$ matrix..........pS3

**Figure S2.** Cyclic voltammograms of blank solutions 0.5 M [TBA][$PF_6$] in $CH_3CN$ solution containing 5% v/v $H_2O$ as a proton source under 100% v/v $CO_2$ and 10% v/v $CO_2$ in a $N_2$ matrix on bare GC electrode and $IM^+_{EE}$ modified electrode..........pS4

**Figure S3.** Cathode potential evolution during during CCE at -3.3 mA $cm^{-2}$ of 2 mM Rh complex and 0.5 M [TBA][$PF_6$] in acetonitrile solution containing 5% v/v $H_2O$ on bare GC and $IM^+_{EE}$ modified cathodes under continuous flow of (a) 100% $CO_2$, (b) 10% $CO_2$, (c) 5% $CO_2$ and (d) 1% $CO_2$..........pS5

**Figure S4.** Cathode potential evolution during CCE at -3.3 mA $cm^{-2}$ of 2 mM Rh complex and 0.5 M [TBA][$PF_6$] in acetonitrile solution containing 5% v/v $H_2O$ under continuous flow of different simulated quasi flue gases on bare GC and $IM^+_{EE}$ modified electrodes..........pS6

**Figure S5.** Cathode potential evolution during CCE at -3.3 mA $cm^{-2}$ of 2 mM Rh complex and 0.5 M [TBA][$PF_6$] in acetonitrile solution containing 5% v/v $H_2O$ under continuous flow of different simulated quasi flue gases on $IM^{+}_{EE}$ modified electrode…...…………………........pS7

**Figure S6.** Linear sweep voltammograms on bare GCE and on IM+EE modified electrode of 2 mM Rh complex and 0.5 M [TBA][PF6] in acetonitrile solution containing 5% v/v $H_2O$ under different gas saturated conditions: Ar, 5% v/v of $CO_2$, $NO_2$ or $SO_2$ in $N_2$ matrix……………pS8

**Figure S7.** $^1$H NMR spectrum of [Rh(bpy)(Cp*)Cl] (300 MHz, $CD_3CN$)…………………...pS9

**Figure S8.** Representative IC chromatograms of a [TBA][$PF_6$] in 5% vol. $H_2O$ in $CH_3CN$, [Rh(bpy)(Cp*)Cl]Cl in the same electrolyte solution, and the catholyte after CCE...……....pS10

**Figure S9.** Calibration curves for $H_2$ and $HCOO^-$ quantification........……………………....pS11

**Table S1**. Rinse test experiment. Two consecutive constant current electrolysis (CCE) on the same bare GCE at -3.3 mA $cm^{-2}$ of 2 mM Rh complex (#1) and with no Rh complex and 0.5 M [TBA][$PF_6$] in $CH_3CN$ solution containing 5% v/v $H_2O$ in a two compartments H-type cell under 100% $CO_2$ stream..........................................................................................................pS12

**Table S2**. Rinse test experiment. Two consecutive constant current electrolysis (CCE) on the same GCE-$IM^{+}_{EE}$ modified electrode at -3.3 mA $cm^{-2}$ of 2 mM Rh complex (#1) and with no Rh complex and 0.5 M [TBA][$PF_6$] in $CH_3CN$ solution containing 5% v/v $H_2O$ in a two compartments H-type cell under 100% $CO_2$ stream……......................................................pS12

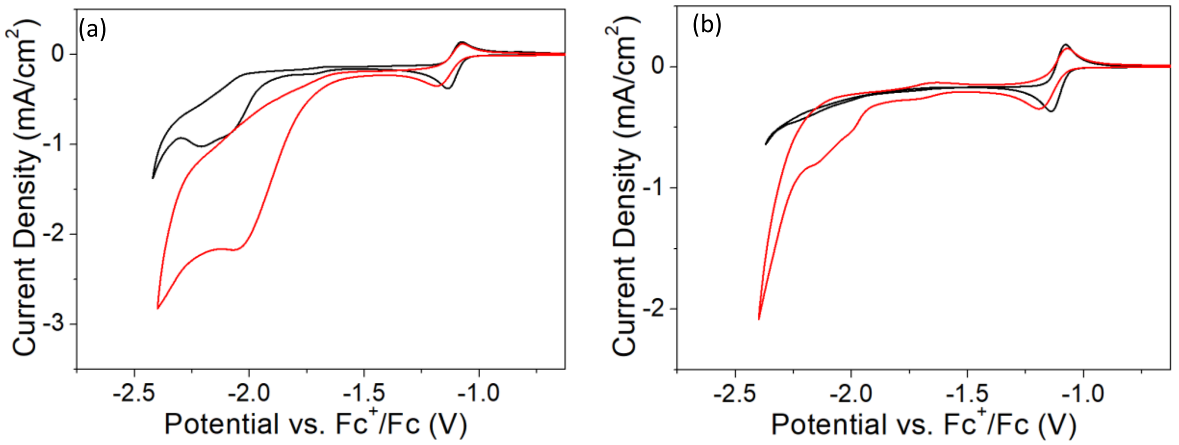


**Figure S1.** Cyclic voltammograms of 2 mM Rh complex and 0.5 M [TBA][$PF_6$] in $CH_3CN$ solution, all containing 5% v/v $H_2O$ as a proton source on bare GC electrode (black plot) and $IM^+_{EE}$ modified electrode (red plot) under different $CO_2$ concentrations. (a) 5% v/v $CO_2$ and (b) 1% v/v $CO_2$ in a $N_2$ matrix. Scan rate 0.01 V $s^{-1}$.

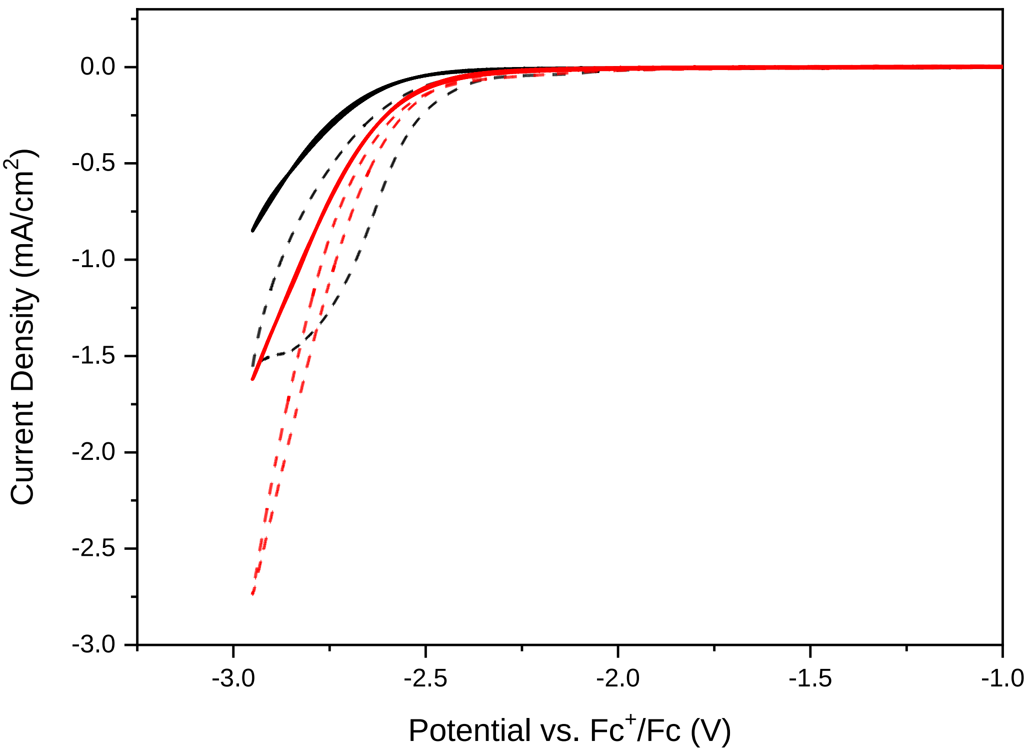


**Figure S2.** Cyclic voltammograms of blank solutions with no Rh complex and 0.5 M [TBA][$PF_6$] in $CH_3CN$ solution containing 5% v/v $H_2O$ as a proton source under 100% v/v $CO_2$ (dashed line) and 10% v/v $CO_2$ in a $N_2$ matrix (solid line) on bare GC electrode (black line) and $IM^+_{EE}$ modified electrode (red line). Scan rate 0.01 V $s^{-1}$.

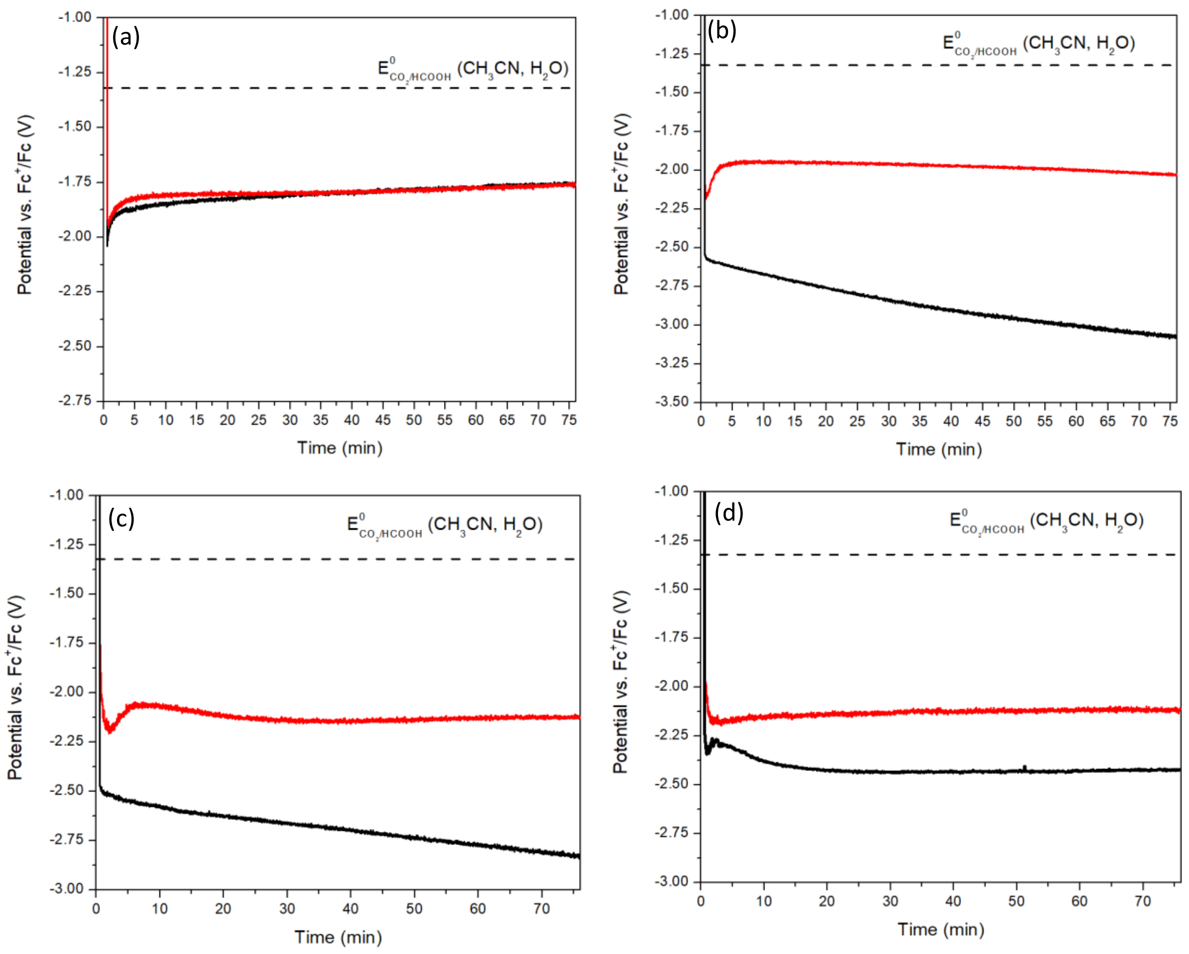


**Figure S3.** Cathode potential evolution during constant current electrolysis (CCE) at -3.3 mA $cm^{-2}$ of 2 mM Rh complex and 0.5 M [TBA][$PF_6$] in acetonitrile solution containing 5% v/v $H_2O$ on bare GC (black plot) and $IM^+_{EE}$ modified cathodes (red plot) under continuous flow of (a) 100% $CO_2$, (b) 10% v/v $CO_2$ in a $N_2$ matrix, (c) 5% v/v $CO_2$ in a $N_2$ matrix and (d) 1% v/v $CO_2$ in a $N_2$ matrix . Total circulated charge 15 C. Solution stirring rate 300 rpm. Continuous gas purging in solution at flow of 14 mL $min^{-1}$.

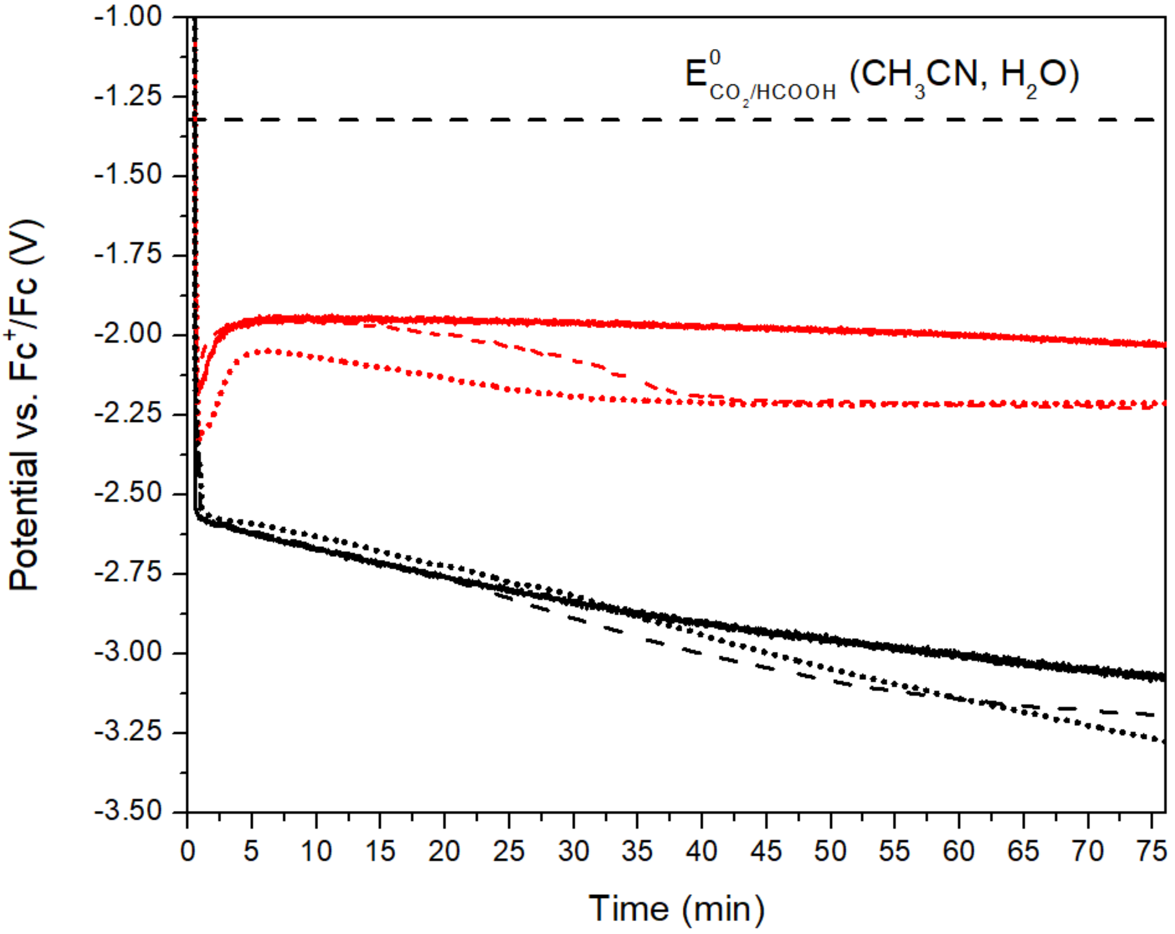


**Figure S4.** Cathode potential evolution during CCE at -3.3 mA cm$^{-2}$ of 2 mM Rh complex and 0.5 M [TBA][$PF_6$] in acetonitrile solution containing 5% v/v $H_2O$ under continuous gas flow (14 mL min$^{-1}$) of different simulated quasi flue gases on bare GC electrode (black plots) and $IM^{+}_{EE}$ modified electrode (red plots). Purged gas: (solid plots) 10% v/v $CO_2$ in $N_2$ matrix, (dashed plots) 10% v/v $CO_2$ and 100 ppm $NO_2$ in $N_2$ matrix and (dotted plots) 10% v/v $CO_2$ and 50 ppm $SO_2$ in $N_2$ matrix. Total circulated charge 15 C. Solution stirring rate 300 rpm.

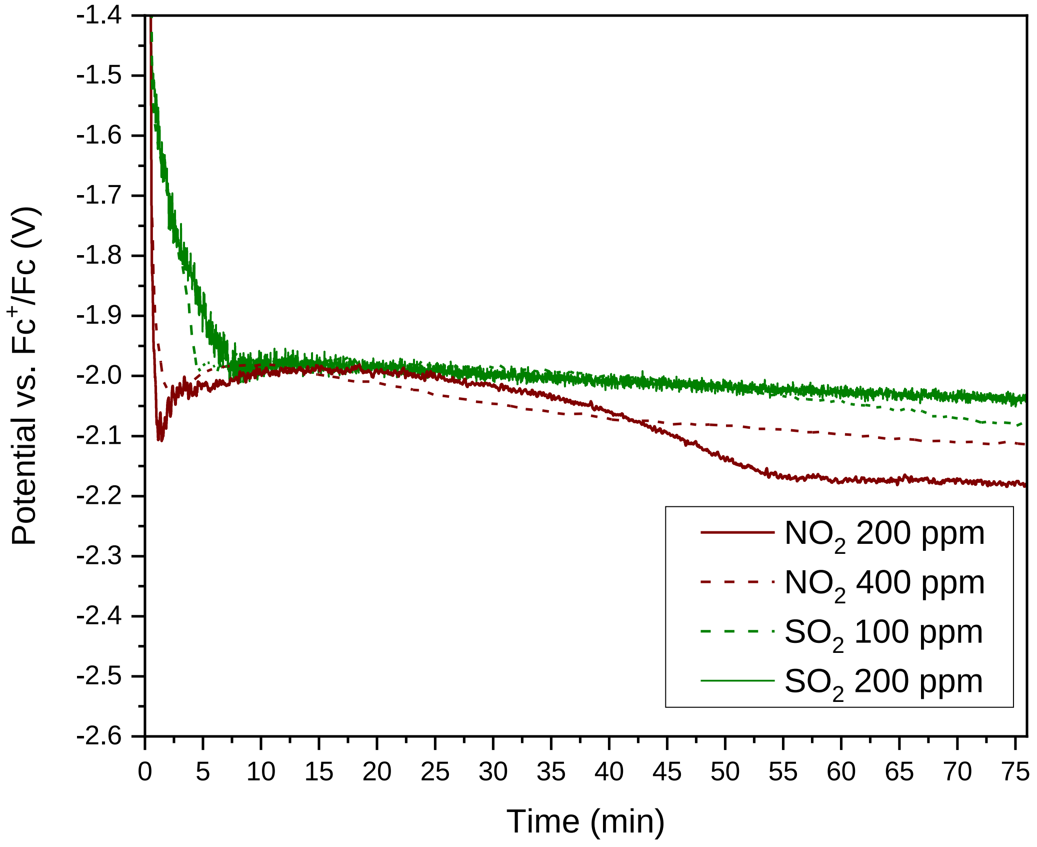


**Figure S5.** Cathode potential evolution during CCE at -3.3 mA cm$^{-2}$ of 2 mM Rh complex and 0.5 M [TBA][$PF_6$] in acetonitrile solution containing 5% v/v $H_2O$ under continuous gas flow (14 mL min$^{-1}$) of different simulated quasi flue gases on $IM^+_{EE}$ modified electrode. Purged gas: 10% v/v $CO_2$ and $NO_2$ in $N_2$ matrix, (brown plots), 10% v/v $CO_2$ and $SO_2$ in $N_2$ matrix (green plots). Total circulated charge 15 C. Solution stirring rate 300 rpm.

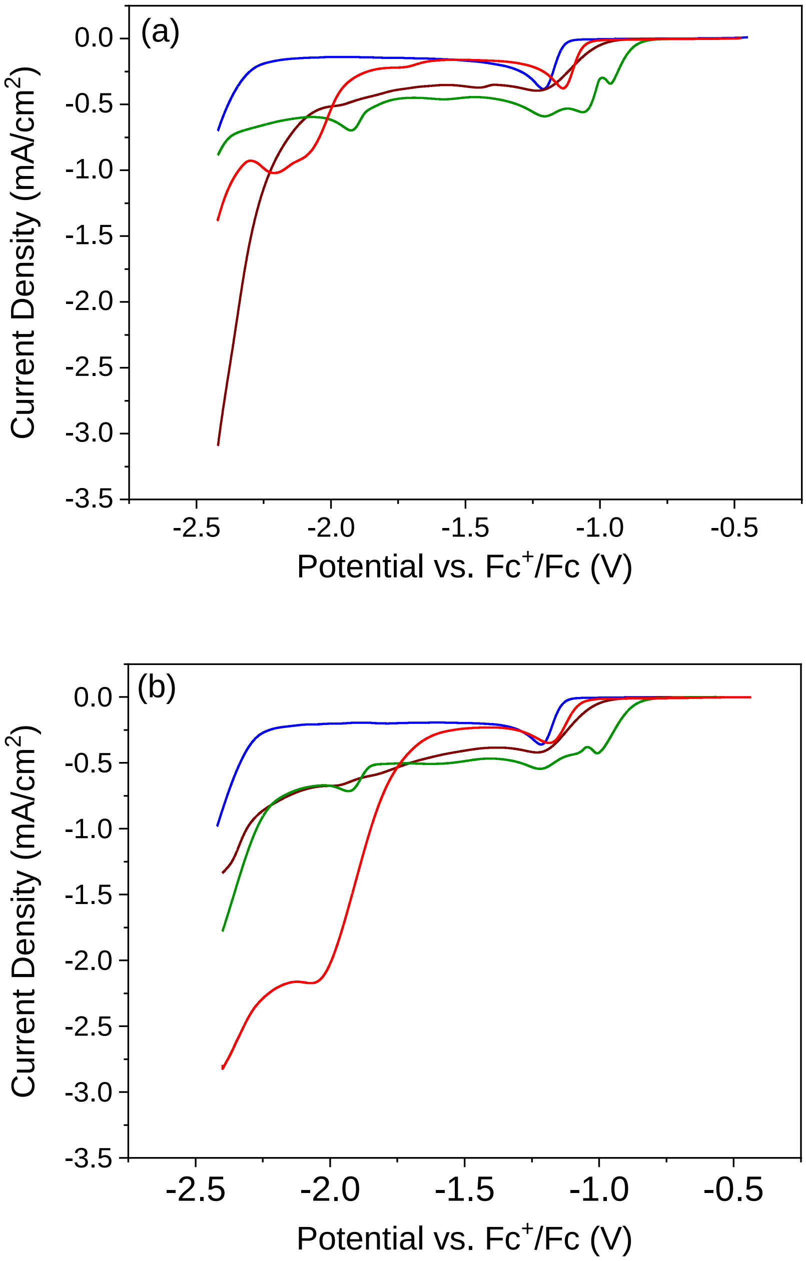


**Figure S6.** Linear sweep voltammograms on bare GCE (a) and on $IM^+_{EE}$ modified electrode (b) of 2 mM Rh complex and 0.5 M [TBA][$PF_6$] in $CH_3CN$ solution containing 5% v/v $H_2O$ under different gas saturated conditions: Ar (blue plot), 5% v/v $CO_2$ in $N_2$ matrix (red plot), 5% v/v $NO_2$ in $N_2$ matrix (brown plot), 5% v/v $SO_2$ in $N_2$ matrix (green plot). Scan rate 0.01 V $s^{-1}$.

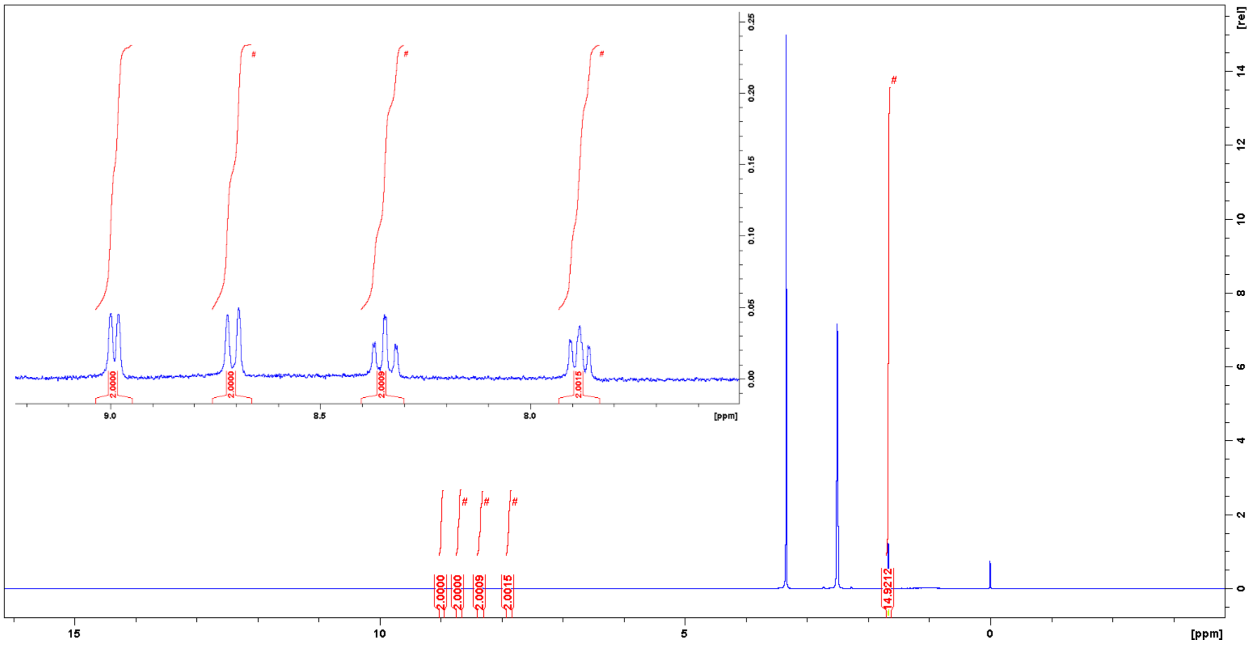


**Figure S7.** $^{1}$H NMR spectrum of [Rh(bpy)(Cp*)Cl] (300 MHz, $CD_3CN$): δ/ppm, 1.66 (s, 15H), 7.88 (dt, J = 6.5 Hz, 2H), 8.34 (dt, J = 7.9 Hz, 2H), 8.70 (d, J = 7.9 Hz, 2H), 8.99 (d, J = 5.4 Hz, 2H).

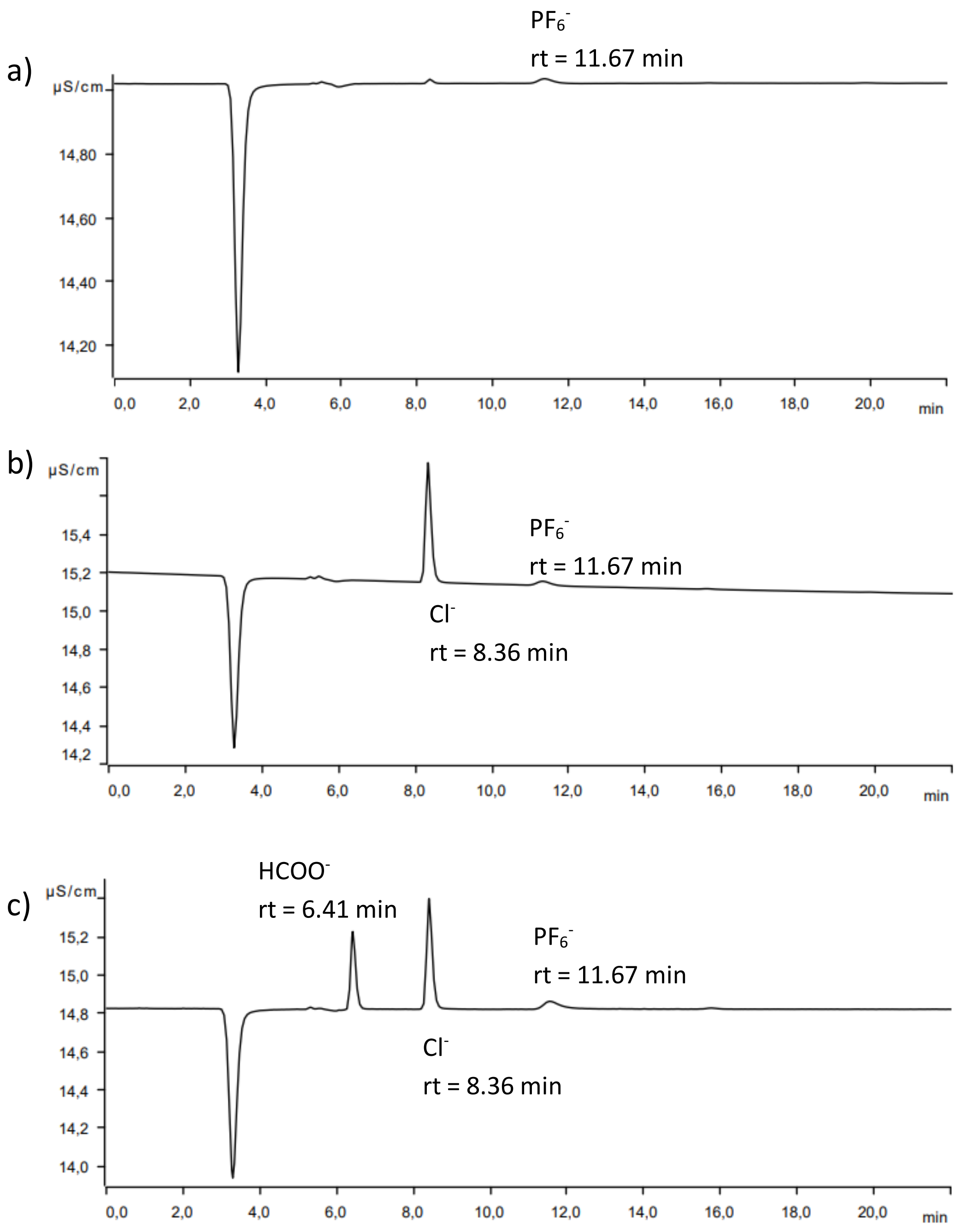


**Figure S8.** Representative IC chromatograms of (a) a blank solution containing 0.5 M [TBA][$PF_6$] in 5% vol. $H_2O$ in $CH_3CN$, (b) a solution containing molecular complex [Rh(bpy)(Cp*)Cl]Cl in the same electrolyte solution, and (c) the catholyte from a bulk electrolysis of 15 C under 100% $CO_2$ using a bare GCE and the molecular Rh complex in solution. Retention times (rt) for formate (6.41 min), chloride (8.36 min) and $PF_6^-$ (11.67 min) are highlighted.

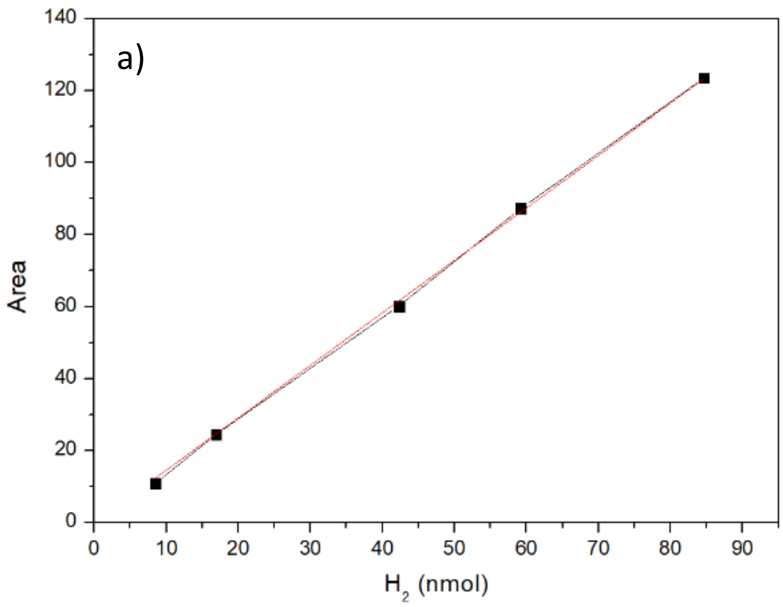


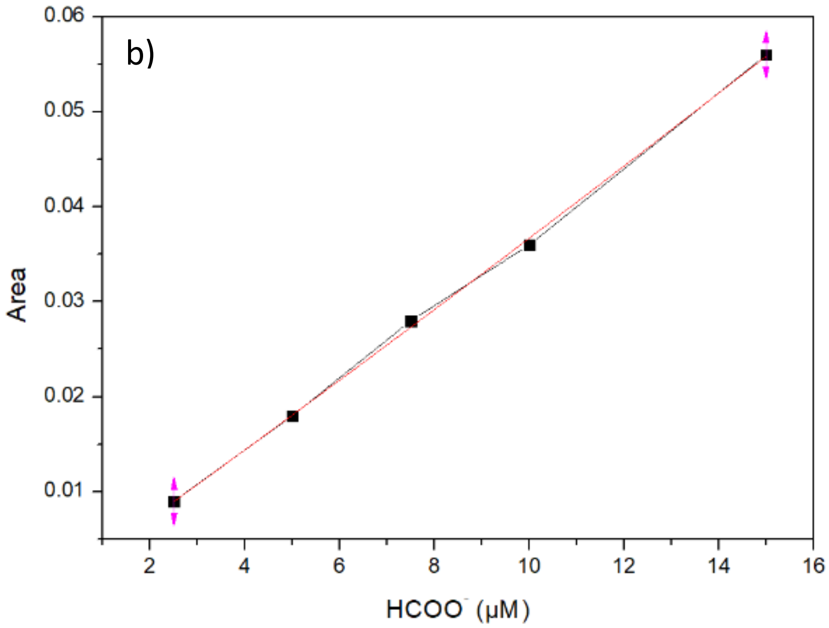


**Figure S9.** Calibration curves for $H_2$ (a) and $HCOO^-$ (b) quantification using gas chromatography and ionic exchange chromatography, respectively. The linear fit for $H_2$ follows the equation y = a + b*x, intercepting at 0 (a = 0), with b = 1.4568 and $R^2$ = 0.9997. For $HCOO^-$, a quadratic fit intercepting at 0 was applied, following the equation y = a + b*x + c*$x^2$, with a = 0, b = 1.718 × $10^{-4}$, c = 5.4099 × $10^{-8}$, and $R^2$ = 0.9998.

**Table S1.** Two consecutive constant current electrolysis (CCE) on the same bare GCE at -3.3 mA $cm^{-2}$ of 2 mM Rh complex (#1) and with no Rh complex (#2) and 0.5 M [TBA][$PF_6$] in $CH_3CN$ solution containing 5% v/v $H_2O$ in a two compartments H-type cell under 100% $CO_2$ saturated atmosphere. Total circulated charge 15 C in each electrolysis. Solution stirring rate 300 rpm.

| **Electrolysis** | $FE_{HCOO-}$ **[%]** | $FE_{H2}$ **[%]** |
|---|---|---|
| **#1** | 86 | 13 |
| **#2** | 17 | 82 |

**Table S2.** Two consecutive constant current electrolysis (CCE) on the same GCE-$IM^{+}_{EE}$ modified electrode at -3.3 mA $cm^{-2}$ of 2 mM Rh complex (#1) and with no Rh complex (#2) and 0.5 M [TBA][$PF_6$] in $CH_3CN$ solution containing 5% v/v $H_2O$ under 100% $CO_2$ saturated atmosphere in a two compartments H-type cell. Total circulated charge 15 C in each electrolysis. Solution stirring rate 300 rpm.

| **Electrolysis** | $\mathbf{FE_{HCOO-}}$ **[%]** | $\mathbf{FE_{H2}}$ **[%]** |
|---|---|---|
| **#1** | 76 | 19 |
| **#2** | 12 | 87 |
